# Sporotrichosis in Mexico

**DOI:** 10.1007/s42770-020-00387-x

**Published:** 2020-10-30

**Authors:** Conchita Toriello, Carolina Brunner-Mendoza, Estela Ruiz-Baca, Esperanza Duarte-Escalante, Amelia Pérez-Mejía, María del Rocío Reyes-Montes

**Affiliations:** 1 Departamento de Microbiología-Parasitología, Facultad de Medicina, Universidad Nacional Autónoma de México (UNAM), 04510 Mexico City, Mexico; 2 Facultad de Ciencias Químicas, Universidad Juárez del Estado de Durango, Av. Veterinaria S/N, 34120 Durango, Mexico

**Keywords:** *Sporothrix* spp., Mexico, Epidemiology, Diagnosis

## Abstract

Sporotrichosis is an endemic mycosis caused by the species of the *Sporothrix* genus, and it is considered one of the most frequent subcutaneous mycoses in Mexico. This mycosis has become a relevant fungal infection in the last two decades. Today, much is known of its epidemiology and distribution, and its taxonomy has undergone revisions. New clinical species have been identified and classified through molecular tools, and they now include *Sporothrix schenckii sensu stricto*, *Sporothrix brasiliensis*, *Sporothrix globosa*, and *Sporothrix luriei*. In this article, we present a systematic review of sporotrichosis in Mexico that analyzes its epidemiology, geographic distribution, and diagnosis. The results show that the most common clinical presentation of sporotrichosis in Mexico is the lymphocutaneous form, with a higher incidence in the 0–15 age range, mainly in males, and for which trauma with plants is the most frequent source of infection. In Mexico, the laboratory diagnosis of sporotrichosis is mainly carried out using conventional methods, but in recent years, several researchers have used molecular methods to identify the *Sporothrix* species. The treatment of choice depends mainly on the clinical form of the disease, the host’s immunological status, and the species of *Sporothrix* involved. Despite the significance of this mycosis in Mexico, public information about sporotrichosis is scarce, and it is not considered reportable according to Mexico’s epidemiological national system, the “Sistema Nacional de Vigilancia Epidemiológica.” Due to the lack of data in Mexico regarding the epidemiology of this disease, we present a systematic review of sporotrichosis in Mexico, between 1914 and 2019, that analyzes its epidemiology, geographic distribution, and diagnosis.

## Introduction

Sporotrichosis is an infection caused by the thermodimorphic fungi of the genus *Sporothrix*. The disease is characterized by nodular lesions in the skin and in the subcutaneous tissue, that subsequently ulcerate, mainly affecting the lymphocutaneous system, but rarely other organs. The transmission pathways are associated with organic matter, animal excreta, or also zoonosis [1–3]. Sporotrichosis is usually acquired through traumatic inoculation with fomites (spines, debris), contaminated soil, and animal scratches [1, 4–6]. Because plant thorns or bushes are often the source of the infection, the disease is commonly known as the “rosebush mycosis” or the “gardener’s mycosis” [6]. Rarely can it be acquired by inhalation of spores and produce a primary lung infection [1, 7]. This mycosis is also widely prevalent in endothermic animals, such as cats, occasionally dogs, armadillos, rats, birds, and parrots, which are a source of zoonotic transmission [5]. Years ago, it has been shown that sporotrichosis can evolve as a severe disseminated disease with visceral and osteoarticular involvement, particularly in individuals with AIDS, individuals receiving immunosuppressive treatments, and other causes of immunodeficiencies, such as diabetes and chronic alcoholism [1, 8].

Sporotrichosis can be classified as cutaneous (which is the most common form) or extracutaneous [1, 6]. However, there are other classification approaches based on the clinical characteristics of the infection, and they are divided as follows: skin (lymphocutaneous, fixed cutaneous, and multiple inoculations), mucous membrane (ocular, nasal, and others), systemic (osteoarticular, disseminated cutaneous, pulmonary, neurological, and other locations/sepsis), and immunoreactive (erythema nodosum, erythema multiforme, Sweet syndrome, and reactive arthritis) [6]. Lymphocutaneous sporotrichosis is the most common form, which predominantly affects the upper extremities (forearm and hands) and the facial region. When there is no dissemination, the form is known as fixed cutaneous sporotrichosis. Ocular sporotrichosis is the most common form among the mucous membrane infections, causing conjunctivitis, episcleritis, uveitis, choroiditis, and retrobulbar lesions. Systemic sporotrichosis is the least frequent of all, and it is mainly associated with individuals with immunosuppression factors such as AIDS, uncontrolled diabetes mellitus, and lymphoma, or individuals under immunosuppressive treatment [1, 6, 8]. Some patients may present a spontaneous resolution of the infection, and there is also an immunoreactive form, in which an exacerbated immune response against the fungus may occur [6].

Although the mycosis is distributed worldwide, most of the cases come from tropical and subtropical areas in Latin America, Africa, and Asia [5]. In Europe, the cases have been recorded intermittently in countries like Italy, Spain, Portugal, the UK, and Turkey [9]. In Latin America, the estimated prevalence rate of sporotrichosis is 0.1 to 0.5%, particularly in Brazil, Colombia, El Salvador, Mexico, Uruguay, and Venezuela, while in Argentina, Ecuador, and Panama, it ranges from 0.01 to 0.02%. In some regions of South America, the disease occurs most frequently during the wet seasons of summer and autumn. In Mexico, the incidence rate increases during the cold and dry seasons, mainly in regions with a temperate and humid climate [5, 10]. There is no substantial evidence about the prevalence of the disease by age or sex, and it is often associated with agriculture, gardening, mining, or other outdoor activities [11].

Sporotrichosis can be diagnosed by a combination of clinical and epidemiological data, and laboratory tests. The transmission of sporotrichosis occurs in open spaces; therefore, other diseases such as cutaneous leishmaniasis, tuberculosis, tularemia, leprosy, and some neoplastic and bacterial lesions should be considered in the differential diagnosis, especially if there are no tools or infrastructure for mycological tests [12, 13].

Traditionally, sporotrichosis is diagnosed considering the results from clinical and laboratory studies. Clinical studies usually provide a presumptive diagnosis, while laboratory procedures are necessary to establish the etiology of the disease [14]. The gold standard diagnosis for sporotrichosis is the culture of clinical samples—in Sabouraud dextrose medium agar (SDA) at 25 to 28 °C—that are obtained from active lesions, pus, secretions, or biopsy. In this medium, the fungus forms filamentous colonies. The typical colony morphology in SDA is a thin mycelium with sessile and sympodial microconidia, while in rich media such as blood and chocolate agar at 37 °C, the fungus forms yeast colonies of elongated blastoconidia [6]. The direct examination of biological samples with 10% potassium hydroxide is useless for the diagnosis of human sporotrichosis, due to the scarcity of fungal elements in the lesions, particularly in the lymphocutaneous and fixed cutaneous forms. However, it is convenient to discard other sporotrichoid skin infections [15]. The histopathological analysis is another alternative method, mainly for disseminated forms [16].

In this article, we present a review of sporotrichosis in Mexico through a systematic revision of articles including the following criteria: the epidemiological data of the patients, such as age, gender, geographic origin, diagnosis, and treatment, published from 1914 to 2019.

## Methods

The databases used in the search were Scopus, PubMed, ScienceDirect, MEDLINE, and SciELO, as well as the archives from the Faculty of Medicine Library, UNAM. The search was performed using the words *Sporothrix*, *Sporothrix schenckii*, and sporotrichosis.

## Results

**Fig. 1:**
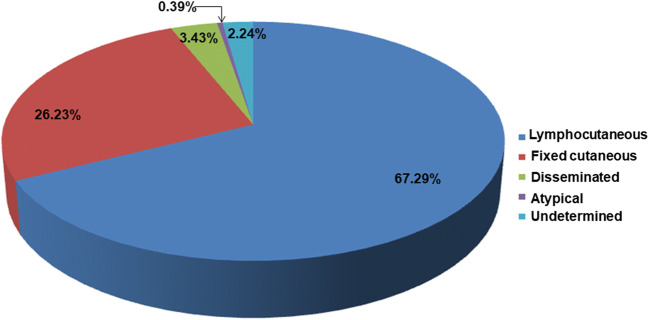
Frequency of clinical presentations of sporotrichosis in Mexico from 1914 to 2019

**Fig. 2:**
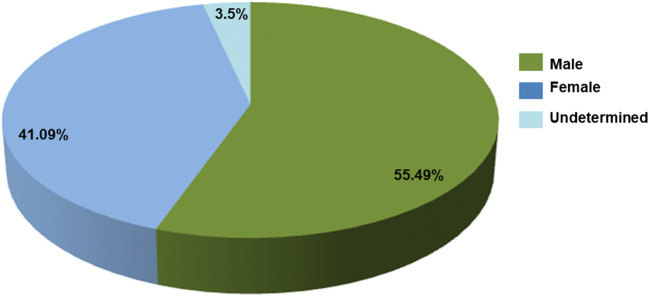
Frequency of sporotrichosis by age groups in Mexico from 1914 to 2019

**Fig. 3:**
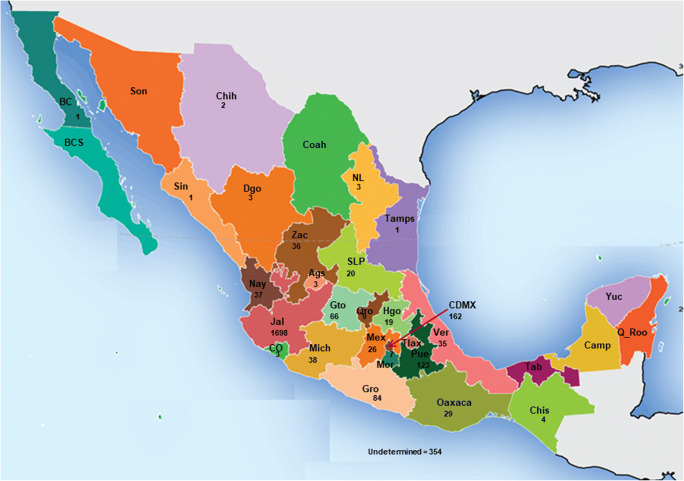
Geographic distribution of sporotrichosis cases in Mexico from 1914 to 2019. Ags, Aguascalientes; BC, Baja California; BCS, Baja California Sur; Camp, Campeche; Coah, Coahuila; Col, Colima; Chis, Chiapas; Chih, Chihuahua; CDMX, Ciudad de México; Dgo, Durango; Gto, Guanajuato; Gro, Guerrero; Hgo, Hidalgo; Jal, Jalisco; Mex, Estado de México; Mich, Michoacán; Mor, Morelos; Nay, Nayarit; NL, Nuevo León; Oaxaca, Oaxaca; Pue, Puebla; Qro, Querétaro; Q_Roo, Quintana Roo; SLP, San Luis Potosí; Sin, Sinaloa; Son, Sonora; Tab, Tabasco; Tamps, Tamaulipas; Tlax, Tlaxcala; Ver, Veracruz; Yuc, Yucatán; Zac, Zacatecas (https://repositoriodocumental.ine.mx)

A total of 40 articles were selected considering the patients’ epidemiological data, such as age, gender, geographic origin, diagnosis, and treatment (Table 1). From these data, 2762 cases with different clinical presentations, such as lymphocutaneous, fixed, disseminated, and atypical sporotrichosis, were found. The most frequent presentation was lymphocutaneous sporotrichosis (67.29%), followed by the fixed (26.23%), the disseminated (3.43%), and the atypical (0.39%) presentations (Fig. 1). Furthermore, according to this revision, there is a higher incidence in males (55.49%), while female individuals showed a lower incidence (41.09%). Regarding age, this review found patients with sporotrichosis in the 0 to > 61 age range, including the most affected group aged 0–15 years (34.15%), followed by other groups aged 16–30 years (16.89%), 31–45 years (12.91%), 46–60 years (14.5%), and, finally, > 61 years (12.69%) (Fig. 2). Moreover, the data obtained from this review showed that the highest number of sporotrichosis cases in Mexico are located, in descending precedence, in Jalisco (*n* = 1698), Mexico City (*n* = 162), Puebla (*n* = 123), Guerrero (*n* = 84), and Guanajuato (*n* = 66). In contrast, in the states of Sonora, Coahuila, Campeche, Baja California Sur, Tabasco, Tlaxcala, Quintana Roo, and Yucatán, there are no sporotrichosis cases recorded until now (Fig. 3). The cases with the highest frequency, found in this study, included students (24.67%), peasants (23.01%), and housewives (19.89%) (Table 2). The laboratory diagnosis of sporotrichosis is mainly carried out using conventional methods (sample culture, isolation of the etiologic agent, macro- and micromorphology, histopathology, and sporotrichin skin test (ST)). Thirty-seven of the articles reviewed included at least one of the aforementioned diagnostic tests, and in 75% of them, immunodiagnostic methods were present; 14 papers reported the use of ST, three used the indirect immunofluorescence assay (IFA), one used the immunodiffusion method, and another one used the Enzyme-Linked Immunosorbent Assay (ELISA) method (Table 1).

**Table 1:** Epidemiological data on sporotrichosis in Mexico

Reference	Cases (*n*)	Age (*n*)	Gender	Geographical origin	Clinical presentation	Source of infection	Culture	Laboratory diagnosis tests	Treatment	Species
Gayón [17]	1	40	F	Ver	L	UD	Positive	Macro- and micromorphology	Potassium iodide	*S. schenckii*
Latapi [18]	1	3	M	Mich	L	UD	Positive	Macro- and micromorphology ST	Potassium iodide	*S. schenckii*
Lavalle [19]	220	0–15 (60) 16–30 (73) 31–45 (31) 46–60 (28) 61 > (25) UD (3)	M (116) F (104)	CDMX (89) Gto(27) Pue (16) Jal (13) Hgo (12) Ver (11) Mex (10) Mich (8) Oaxaca (8) SLP (6) Zac (4) Qro (4) Gro (4) Mor (3) Ags (1) Col (1) Dgo (1) Chis (1) Tamps (1)	L (129) F (62) D (11) A (6) UD (12)	UD	Positive (135)	Macro- and micromorphology ST Biopsy	Amphotericin B Potassium iodide	*S. schenckii*
Mayorga et al. [20]	822	< 1–15 (239) 16–30 (154) 31–45 (114) 46–60 (132) > 61 (102) UD (81)	M (478) F (344)	Jal (539) Nay (10) Zac (9) Mich (4) Gto (1) UD (259)	L (567) F (184) D (10) UD (61)	UD	UD	UD	UD	UD
Espinosa-Texis et al. [21]	50	< 1–15 (239) 16–30 (154) 31–45 (114) 46–60 (132) > 61 (102) UD (81)	M (31) F (19)	Pue (16) CDMX (14) Jal (8) Oaxaca (4) Gro (3) Hgo (2) Gto (1) Ags (1) Zac (1)	L (41) F (8) D (1)	UD	Positive (47/50)	ST Histopathology IFA	UD	*S. schenckii* (94%)
Padilla et al. [22]	1	63	M	CDMX	L	UD	Positive	Direct exam (+) X-ray	Potassium iodide	*S. schenckii*
Padilla and Saucedo [23]	1	22	M	Pue	D	UD	Positive	ST X-ray Histopathology	Potassium iodide	*S. schenckii*
Padilla et al. [24]	1	18	M	Hgo	F	UD	Positive	Direct exam (+) Histopathology ST X-ray	Potassium iodide	*S. schenckii*
Padilla et al. [25]	1	62	F	Pue	L	UD	Positive	Histopathology	Potassium iodide	*S. schenckii*
Vega-Morquecho et al. [26]	1	58	F	Gto	D	UD	Positive	Direct exam (+) ST Histopathology X-ray	Potassium iodide Itraconazole	*S. schenckii*
Padilla et al. [27]	120 (retrospective study 1956–2003)	Infants (6) Preschoolers (20) Schoolers (39) Adolescents (55)	M (61) F (59)	CDMX (38) Mex (15) Pue (14) Ver (14) Gto (11) Oaxaca (6) Hgo (4) Chis (3) Mich (3) Mor (3) Qro (3) Gro (2) SLP(2) Jal (1) Zac (1)	L (98) F (22)	UD	Positive (120)	UD	UD	UD
Méndez-Tovar et al. [28]	1	68	M	Mich	F	UD	Positive	Direct exam. (+) Histopathology	Itraconazole	*S. schenckii*
Poletti et al. [29]	4	7	M	Jal	F	Cat scratch	Positive	Direct exam. (+)	Potassium iodide Itraconazole	*S. schenckii*
		9	M	Jal	F	UD	Positive	Histopathology	Itraconazole	*S. schenckii*
		9	M	Oaxaca	F	UD	Positive	Direct exam (+) ST	UD	*S. schenckii*
		11	M	Ags	F	Squirrel bite	Positive	Histopathology	Itraconazole	*S. schenckii*
Carrada-Bravo [30]	5	1. 9	F	Gto	L	UD	Positive	Histopathology	Potassium iodide	*S. schenckii*
		2.9	F	Gto	L	UD	Positive	ID IFA	Itraconazole	*S. schenckii*
		5	F	Gto	L	UD	Positive	ID IFA	Potassium iodide	*S. schenckii*
		9	M	Gto	F	UD	Positive	UD	Potassium iodide	*S. schenckii*
		13	F	Gto	F	UD	Positive	UD	Potassium iodide	*S. schenckii*
Macotela-Ruiz and Nochebuena-Ramos [31]	55	0–15 (17) 16–30 (13) 31–45 (3) 46–60 (12) > 61 (10)	M (33) F (22)	Pue	L (31) F (17) D (4) A (3)	UD	Positive (55)	Macro- and micromorphology ST	Potassium iodide Itraconazole Ketoconazole	*S. schenckii*
Muñoz-Estrada et al. [32]	1	12	M	Sin	L	UD	Positive	Macro- and micromorphology	Itraconazole	*S. schenckii*
Carrada-Bravo [33]	1	42	M	UD	A	UD	Positive	ST ELISA IFA	Itraconazole	*S. schenckii*
Padilla et al. [34]	1	13	M	Oaxaca	L	Trauma with plants	Positive	Macro- and micromorphology ST Histopathology	Potassium iodide	*S. schenckii*
Padilla et al. [35]	1	54	F	Oaxaca	L	UD	Positive	Macro- and micromorphology Direct exam (+) ST	Potassium iodide	*S. schenckii*
Arenas et al. [36]	13	72	M	Mich	L	UD	Positive	UD	UD	UD
		72	F	Pue	L	UD	Positive	UD	UD	*S. schenckii*
		60	F	SLP	F	UD	Positive	UD	UD	*S. schenckii*
		63	F	CDMX	L	UD	Positive	UD	UD	*S. schenckii*
		60	F	Gto	L	UD	Positive	UD	UD	*S. schenckii*
		55	M	Gto	L	UD	Positive	UD	UD	*S. schenckii*
		76	M	Gto	D	UD	Positive	UD	UD	*S. schenckii*
		75	F	Gro	D	UD	Positive	UD	UD	*S. schenckii*
		64	M	Gto	L	UD	Positive	UD	UD	*S. schenckii*
		24	M	Pue	D	UD	Positive	UD	UD	*S. schenckii*
		9	M	Oaxaca	L	UD	Positive	UD	UD	*S. schenckii*
		35	M	Gto	D	UD	Positive	UD	UD	*S. schenckii*
		12	M	Dgo	L	UD	Positive	UD	UD	*S. schenckii*
Bada del Moral et al. [37]	5	58	F	Ver	L	UD	Positive	Biopsy H-E	Potassium iodide	*S. schenckii*
		32	M	Ver	F	Ant bite	Positive	Histopathology H-E	Potassium iodide	*S. schenckii*
		25	M	Ver	L	UD	Positive	Histopathology H-E	Potassium iodide	*S. schenckii*
		26	M	Ver	F	Motorcycle accident	Positive	Histopathology	Potassium iodide	UD
		75	F	Ver	L	UD	Positive	Histopathology PAS	UD	*S. schenckii*
Bonifaz et al. [38]	25	0.8–17.5	UD	UD	L (16) F (8) D (1)	Trauma with plants (17) Squirrel scratch (2) Squirrel bite (1) Cat scratch (1) Rat bite (1)	Positive (24/25)	Macro- and micromorphology Dimorphism ST Biopsy H-E and PAS	Potassium iodide Itraconazole	*S. schenckii*
Chávez et al. [39]	1	78	F	UD	D	Excoriation on lip from fall	Positive	Histopathology PAS	Amphotericin B	*S. schenckii*
Fonseca-Reyes et al. [40]	1	40	M	UD	D	UD	Positive	Histopathology PAS	Amphotericin B	*S. schenckii*
Munguía et al. [41]	10	62	F	Pue	L	Trauma with plants	Positive	ST Reproduction of sporotrichosis in mice	UD	*S. schenckii*
		44	F	Pue	L	Trauma with plants	Positive	ST Reproduction of sporotrichosis in mice	UD	*S. schenckii*
		49	M	Pue	F	Trauma with plants	Positive	ST Reproduction of sporotrichosis in mice	UD	*S. schenckii*
		39	M	Pue	L	Trauma with plants	Positive	ST Reproduction of sporotrichosis in mice	UD	*S. schenckii*
		27	F	Pue	L	Trauma with plants	Positive	ST	UD	*S. schenckii*
		34	F	Pue	L	Trauma with plants	Positive	ST Reproduction of sporotrichosis in mice	UD	*S. schenckii*
		19	F	Pue	L	Trauma with plants	Positive	ST Reproduction of sporotrichosis in mice	UD	*S. schenckii*
		78	F	Pue	F	Trauma with plants	Positive	ST Reproduction of sporotrichosis in mice	UD	*S. schenckii*
		19	F	Pue	F	Trauma with plants	Positive	ST Reproduction of sporotrichosis in mice	UD	*S. schenckii*
		29	M	Pue	F	Trauma with plants	Positive	ST Reproduction of sporotrichosis in mice	UD	*S. schenckii*
García-Vargas et al. [42]	133	< 15	M (76) F (57)	Nay (4) Jal (75) UD (54)	L (72/133) F (58/133) D (3/133)	UD	Positive (133)	Macro- and micromorphology	UD	*S. schenckii*
Barba-Borrego et al. [43]	1	12	M	UD	L	UD	Positive	Macro- and micromorphology	Potassium iodide	*S. schenckii*
Roldán-Marín et al. [44]	1	53	F	UD	F	UD	Negative	Histopathology ST	Potassium iodide	*S. schenckii*
Gutierrez-Morales et al. [45]	1	39	M	Ver	A	UD	Positive	Histopathology	Potassium iodide antifungal UD	*S. schenckii*
Romero-Cabello et al. [46]	1	36	M	Pue	D	UD	Positive	Histopathology Wright and Giemsa ST	Itraconazole Potassium iodide Amphotericin B	*S. schenckii* (*sensu stricto*)
Rojas-Padilla et al. [47]	1	13	M	Oaxaca	L	Spider bite	Positive	Macro- and micromorphology ST	Potassium iodide	*S. schenckii*
Chávez López et al. [48]	1	36	F	Gro	D	UD	Positive	macro and micromorphology Biopsy	Potassium iodide	*Sporothrix* spp.
Palma-Ramos et al. [49]	11	UD	UD	UD	F	UD	UD	Biopsy	UD	*Sporothrix* spp.
Cotino Sánchez et al. [50]	1	68	M	Dgo	D	UD	Positive	Biopsy	Itraconazole	*Sporothrix* spp.
Estrada-Castañón et al. [51]	73	Children (37) Adults (36)	M (33) (45.2%) F (40) (54.8%)	Gro	L (41) F (24) D (8)	UD	Positive	Macro- and micromorphology (73) ST (44) Biopsy (29)	Potassium iodide	*Sporothrix* spp. *S. schenckii*
Rojas et al. [52]	39	UD	UD	CDMX (17) SLP (3) Col (2) NL (3) Pue (4) Oaxaca (6) Jal (2) Ver (2)	L (36) F (2) D (1)	UD	Positive	Macro- and micromorphology Analysis of the partial sequence of the calmodulin gene	UD	*S. schenckii* (38) *S. globose (1)*
Rangel-Gamboa et al. [53]	22	UD		SLP (8) Jal (1) Zac (1) Pue (3) Mich (1) CDMX (2) Qro (2) Gto (2) Mor (1) Mex (1)	L (17) F (4) D (1)	Trauma with plant (*Crataegus pubescens*) (1) Rodent bite (1) UD (20)	Positive	Analysis of the partial sequences of the calmodulin and calcium/calmodulin-dependent kinase genes	UD	*S. schenckii* (19) *S. globosa* (4)
Ochoa-Reyes et al. [54]	1	84	F	Chih	F	UD	Positive	Macro- and micromorphology Analysis of the partial sequence of the calmodulin gene	Itraconazole	*S. schenckii* (sensu *lato*)
Puebla-Miranda et al. [55]	1	23	M	BC	L	Trauma with rock	Positive	Macro- and micromorphology	Potassium iodide	*S. schenckii* (sensu stricto)
Mayorga-Rodriguez et al. [10]	1134	< 1–15 (292) 16–30 (199) 31–45 (156) 46–60 (200) ≥ 61 (199) UD (88)	M (669) F (465)	Jal (1057) Nay (23) Zac (20) Mich (19) Gto (13) Ver (1) Chih (1)	L (782) F (308) D 44	UD	UD	UD	UD	*Sporothrix* complex

*IFA*, indirect immunofluorescence assay; *UD*, undetermined; *ST*, skin test; *ID*, immunodiffusion; *PAS*, periodic acid-Schiff stain; *H-E*, hematoxylin-eosin stain; *M*, male; *F*, female; *L*, lymphocutaneous; *F*, fixed; *D*, disseminated; *A*, atypical; *Ags*, Aguascalientes; *BC*, Baja California; *Col*, Colima; *Chis*, Chiapas; *Chih*, Chihuahua; *CDMX*, Ciudad de México; *Dgo*, Durango; *Gto*, Guanajuato; *Gro*, Guerrero; *Hgo*, Hidalgo; *Jal*, Jalisco; *Mex*, Estado de México; *Mich*, Michoacán; *Mor*, Morelos; *Nay*, Nayarit; *NL*, Nuevo León; *Oaxaca*, Oaxaca; *Pue*, Puebla; *Qro*, Querétaro; *SLP*, San Luis Potosí; *Sin*, Sinaloa; *Tamps*, Tamaulipas; *Ver*, Veracruz; *Zac*, Zacatecas

**Table 2:** Occupational activities of patients with sporotrichosis

Activities	Number of cases (*n* = 2764)	Frequency (%)
Student	682	24.67
Farmer	636	23.01
Housewife	550	19.89
Employee	87	3.14
Builder	40	1.44
Merchant	31	1.12
Gardener	28	1.01
Carpenter	14	0.50
Professional	13	0.47
Mechanic	8	0.28
Florist	5	0.18
Painter	5	0.18
Poultry man	3	0.10
Railway man	1	0.03
Indigent	1	0.03
Undetermined	660	23.87

Finally, the revision evidenced that the most frequently employed treatment for sporotrichosis in Mexico is potassium iodide, even though the treatment of choice (itraconazole, terbinafine, amphotericin B, and others) mainly depends on the clinical form of the disease, the host’s immunological status, and the species of *Sporothrix* involved (Table 1).

## Discussion

Sporotrichosis is considered one of the most frequent subcutaneous mycoses in Mexico. For a long time, it ranked second after mycetoma, but most of the cases reported for mycetoma are caused by *Nocardia brasiliensis* and not by fungal species [56].

Despite the significance of this mycosis in Mexico, public information about sporotrichosis is scarce, and it is not considered a reportable disease according to the Mexican epidemiological national system, the “Sistema Nacional de Vigilancia Epidemiológica” [57].

Several studies consider lymphocutaneous and fixed sporotrichosis as the most frequent forms. The results of this review showed that the lymphocutaneous sporotrichosis is the most frequent form in Mexico. The possibility that the immune system of each individual, or the species or strain of *Sporothrix* is related to the clinical presentation is a hypothesis that is still under discussion and study [13].

Regarding the prevalence by gender or age, our results show that the most affected age group is between 0 to 15 years. These results are in accordance with those obtained in a retrospective study conducted by Ramírez-Soto et al. [58] in Peru, which showed that 62% of the cases of sporotrichosis involved children under 14 years of age. Our results are also consistent with the findings from another epidemiology study done in Venezuela, where 34.5% of the sporotrichosis cases diagnosed included patients aged < 15 years [59]. However, the infection depends on the exposure to the fungus, and it is more related to specific occupational and recreational activities in each country. As observed in this review, the highest frequency of the disease was recorded in students, possibly due to their participation in outdoor recreational activities, during which they may suffer from trauma involving material contaminated by the fungus. In India and Japan, there is a higher prevalence of sporotrichosis in females, due to their role in agricultural activities [60]. Likewise, in Brazil, females are most frequently infected, either by zoonotic transmission or by trauma with thorns or bushes [61, 62]. On the other hand, in South Africa, the incidence rate in males is higher than that in females, with a 3:1 ratio, because males participate more frequently in outdoor activities and activities related to mining [63]. Lastly, in Asian countries, such as China and India, sporotrichosis is more common in females than in males [64].

In this review, most of the cases did not offer information about the source of infection. A few cases, in which this data was reported, were attributed to skin trauma with plant sticks, cat scratches, bites and scratches from squirrels, rat bites, trauma with debris, and spider bites. In the 40 studies analyzed, the most frequently reported source of infection was skin trauma with plant sticks (Table 1). It is worth mentioning that most of the areas where sporotrichosis cases occur are temperate, forested mountainous areas, with altitudes of approximately ± 2000 m.a.s.l. and summer rains. According to a meta-analysis performed by Ramirez-Soto et al. [58], *Sporothrix* spp. have specific ecological niches within endemic areas, and they grow in soils between 6.6 and 28.84 °C, and a relative humidity between 37.5 and 99.06%.

Concerning the diagnosis of sporotrichosis, phenotypic identification requires 5 to 7 days for culture growth and an additional 10 to 21 days for the physiological test [14]. In Mexico, paraclinical diagnosis of sporotrichosis is mainly carried out using conventional methods (sample culture, isolation of the etiologic agent, macro- and micromorphology, histopathology, and ST); thus, most of the records used for this review considered *S. schenckii* as the only etiological agent.

Immunodiagnostic methods have emerged as an alternative for the diagnosis of sporotrichosis. At first, precipitation and agglutination methods were used [65], but recently, immunoenzymatic assays have been considered new options [66, 67]. These tests are based on the use of antigens obtained from epitopes located on the surface of the *Sporothrix* cell wall, related to N- and O-linked oligosaccharides of peptidorhamnomannan [68, 69], where the O-linked pentasaccharide has been the primary epitope identified in the peptidorhamnomannan fraction [69]. Although purified antigens have been obtained for serological tests, they have significant limitations, such as low reproducibility and cross-reactivity. Until now, no immunological method has allowed for the identification of *Sporothrix* at the species level. Another immunological method widely used in epidemiological studies is the skin test (ST) with sporotrichin. This test determines if the patient has been in contact with the etiologic agent [70].

In recent years (since 2014), several researchers have used molecular methods to identify *Sporothrix* species [53, 71–73]. The most informative loci used for species recognition are located in regions encoding proteins such as calmodulin [74, 75], beta-tubulin [74, 76, 77], the Translation Elongation Factor [4, 64, 77], and the “fungal barcoding” regions (the ribosomal internal transcribed spacers) [78, 79]. Several molecular techniques, as the nested PCR [80, 81]; the Random Amplification of Polymorphic DNA (RAPD) [82]; the Restriction Fragment Length Polymorphism (RFLP) [83]; the Random Amplified Polymorphic DNA (RAPD) [84]; the Amplified Fragment Length Polymorphism (AFLP) [85], and the Rolling Circle Amplification (RCA) [86], have been used successfully. However, the end-point PCR and the real-time multiplex PCR, using fluorescent probes to identify *S. globosa*, *S. schenckii*, and *S. brasiliensis*, predominate [87].

Molecular tools have shown that *Sporothrix* is a complex fungus formed by phylogenetically related cryptic species, some of them considered of medical relevance, such as *S. brasiliensis*, *S. schenckii* *sensu stricto*, *S. globosa*, *S. mexicana*, *S. luriei*, and *S. pallida* [6, 88]. New evidence derived from a population genetic analysis of Mexican native isolates has shed light on an indeterminate clade within *S. schenckii*, which is the species involved in most of the sporotrichosis cases in Mexico [72]. As for *S. globose* and *S. schenckii sensu lato* and *sensu stricto*, they have a worldwide distribution [5]. In Asian countries, *S. globosa* is the predominant endemic species, with a prevalence of 99.3% [64], while in Brazil, *S. brasiliensis* has displaced *S. schenckii* as the most prevalent species [89].

In vitro studies have shown that *Sporothrix* species differ in virulence and antifungal susceptibility [75, 90], suggesting that the combination of different antifungals can generate a favorable response [91]. Potassium iodide and/or itraconazole (ITC) are the first-line treatments for fixed cutaneous and lymphocutaneous sporotrichosis [92]. Terbinafine has been considered the second-line treatment for lymphocutaneous and cutaneous sporotrichosis, in addition to being an excellent therapeutic option for patients with contraindications to the use of itraconazole or potassium iodide [6, 93]. Amphotericin B is used in the disseminated, systemic, pulmonary, and osteoarticular forms [92, 94]. In pregnant or lactating women with fixed cutaneous sporotrichosis, the use of local thermotherapy (42–43 °C) is recommended, due to the thermolability of the fungus, and amphotericin B is recommended in severe cases. Immunosuppressed patients generally require life-long suppressive therapy [95]. The duration and treatment are based on the “Clinical Practice Guidelines for the Management of Sporotrichosis: 2007 Update by the Infectious Diseases Society of America” [94].

## Conclusions

In Mexico, sporotrichosis has proven to be one of the most frequent subcutaneous mycoses. This study showed that the states with the highest number of cases are Jalisco, Mexico City, Puebla, Guerrero, and Guanajuato. The highest incidence of cases is reported in males, in the ≥ 0–15 age range, showing a lymphocutaneous clinical presentation, and for whom plant traumatisms is identified as the main source of infection. The commonly identified species were *S. schenckii* and *S. globosa*. The most frequently used treatment for sporotrichosis is potassium iodide. We emphasize that this is the first retrospective study in Mexico that has done an analysis of sporotrichosis’ epidemiology, geographic distribution, and diagnosis reported between 1914 and 2019. This knowledge could be used to aid in the adoption of strategic public health policies aimed at controlling epidemics.
